# Privacy-Preserving Collaborative Diabetes Prediction in Heterogeneous Health Care Systems: Algorithm Development and Validation of a Secure Federated Ensemble Framework

**DOI:** 10.2196/79166

**Published:** 2026-01-26

**Authors:** Md Rakibul Hasan, Juan Li

**Affiliations:** 1 Department of Computer Science, Faculty, North Dakota State University, 258 Quentin Burdick Bldg, Computer Science Department, NDSU, 1320 Albrecht Blvd, Fargo, ND, 58105, United States, 1 701-231-9662

**Keywords:** blockchain, decentralized health care, diabetes prediction, ensemble learning, federated learning, knowledge distillation, privacy-preserving AI, artificial intelligence, AI

## Abstract

**Background:**

Diabetes prediction requires accurate, privacy-preserving, and scalable solutions. Traditional machine learning models rely on centralized data, posing risks to data privacy and regulatory compliance. Moreover, health care settings are highly heterogeneous, with diverse participants, hospitals, clinics, and wearables, producing nonindependent and identically distributed data and operating under varied computational constraints. Learning in isolation at individual institutions limits model generalizability and effectiveness. Collaborative federated learning (FL) enables institutions to jointly train models without sharing raw data, but current approaches often struggle with heterogeneity, security threats, and system coordination.

**Objective:**

This study aims to develop a secure, scalable, and privacy-preserving framework for diabetes prediction by integrating FL with ensemble modeling, blockchain-based access control, and knowledge distillation. The framework is designed to handle data heterogeneity, nonindependent and identically distributed distributions, and varying computational capacities across diverse health care participants while simultaneously enhancing data privacy, security, and trust.

**Methods:**

We propose a federated ensemble learning framework, FedEnTrust, that enables decentralized health care participants to collaboratively train models without sharing raw data. Each participant shares soft label outputs, which are distilled and aggregated through adaptive weighted voting to form a global consensus. The framework supports heterogeneous participants by assigning model architectures based on local computational capacity. To ensure secure and transparent coordination, a blockchain-enabled smart contract governs participant registration, role assignment, and model submission with strict role-based access control. We evaluated the system on the PIMA Indians Diabetes Dataset, measuring prediction accuracy, communication efficiency, and blockchain performance.

**Results:**

The FedEnTrust framework achieved 84.2% accuracy, with precision, recall, and *F*_1_-score of 84.6%, 88.6%, and 86.4%, respectively, outperforming existing decentralized models and nearing centralized deep learning benchmarks. The blockchain-based smart contract ensured 100% success for authorized transactions and rejected all unauthorized attempts, including malicious submissions. The average blockchain latency was 210 milliseconds, with a gas cost of ~107,940 units, enabling secure, real-time interaction. Throughout, patient privacy was preserved by exchanging only model metadata, not raw data.

**Conclusions:**

FedEnTrust offers a deployable, privacy-preserving solution for decentralized health care prediction by integrating FL, ensemble modeling, blockchain-based access control, and knowledge distillation. It balances accuracy, scalability, and ethical data use while enhancing security and trust. This work demonstrates that secure federated ensemble systems can serve as practical alternatives to centralized artificial intelligence models in real-world health care applications.

## Introduction

Diabetes continues to pose a growing global health burden, requiring timely prediction and proactive management to reduce complications and improve quality of life [1]. While machine learning has emerged as a powerful tool for diabetes prediction, conventional approaches often rely on centralized data repositories [2-4]. This reliance introduces serious challenges related to patient privacy, regulatory compliance (eg, Health Insurance Portability and Accountability Act (HIPAA), General Data Protection Regulation (GDPR), and susceptibility to cyberattacks [5]. Moreover, centralized data aggregation is increasingly impractical due to fragmented data ownership across institutions and regions [6].

Real-world health care systems are inherently heterogeneous, encompassing a wide range of contributors—from large hospitals and urban clinics to wearable health devices in remote settings [7]. These entities vary significantly in data volume, quality, and computational capacity. The data are often nonindependent and identically distributed (non-IID), reflecting demographic, clinical, and behavioral diversity [8]. As a result, models trained within a single institution or on homogeneous datasets often struggle to generalize across settings, limiting their effectiveness and scalability.

To address these limitations, collaborative federated learning (FL) has emerged as a compelling solution [9]. However, applying FL to real-world diabetes prediction presents several unresolved challenges. In particular, current FL frameworks often struggle with:

- security vulnerabilities, such as model poisoning and adversarial manipulation [10]
- lack of coordination and trust, especially in decentralized, multiparty settings [11]
- performance degradation due to client heterogeneity and non-IID data distributions [12]

While several FL frameworks [13-16] have been explored for decentralized health care analytics, most assume homogeneous model architectures, single global models, or idealized trust environments and do not explicitly address lightweight or resource-constrained participants at the edge [17,18]. Existing systems, such as Biscotti [19] and Chang et al [20], rely on gradient sharing and therefore require structurally aligned models and consistent computational resources, while recent blockchain-enabled FL frameworks incorporate differential privacy but still assume homogeneous models or centralized coordination [21,22]. Furthermore, blockchain [23], a promising technology for ensuring integrity, transparency, and access control in decentralized systems, has seen limited integration with FL, especially in diabetes prediction contexts. Other blockchain-enabled approaches, such as Shalan et al [24], provide secure access control but do not incorporate mechanisms for interoperable knowledge sharing across heterogeneous local models.

In contrast, FedEnTrust introduces an integrated design that simultaneously addresses model heterogeneity, non-IID data, trust and identity verification, and secure update submission. By combining soft-label knowledge distillation with blockchain-verified RBAC, FedEnTrust enables robust collaboration across diverse health care systems while preventing unauthorized or malicious updates. FedEnTrust introduces a novel integration of:

- ensemble learning, allowing clients to train diverse local models best suited to their data and computational constraints
- soft-label knowledge distillation, enabling effective model aggregation across non-IID participants
- blockchain-based smart contracts, which provide tamper-proof coordination, role-based access control, and participant accountability

FedEnTrust represents a step forward in secure and collaborative artificial intelligence (AI) for health care, with the following key contributions:

1. Heterogeneity-aware ensemble design: Each participant trains a model tailored to its resource level, supporting real-world deployment across varied health care nodes.
2. Knowledge distillation-based aggregation: We introduce a soft-label ensemble mechanism that improves convergence and generalization across non-IID data.
3. Blockchain-enabled trust layer: Our smart contract system enforces participant registration, access control, and secure model submissions without a centralized authority.
4. Comprehensive evaluation: Using the PIMA Indians Diabetes Dataset, we demonstrate that FedEnTrust improves prediction accuracy; maintains privacy; and ensures secure, low-latency collaboration.

By addressing the intersection of privacy, trust, heterogeneity, and security, FedEnTrust provides a practical and deployable framework for AI-powered diabetes prediction in real-world, decentralized health care systems.

## Methods

### Overview of FedEnTrust

FedEnTrust is a secure, privacy-preserving federated ensemble learning framework designed to address the challenges of decentralized diabetes prediction across heterogeneous health care environments. It enables collaborative learning without centralizing sensitive patient data, accommodates diverse computational resources, and defends against malicious behaviors through a blockchain-coordinated trust infrastructure. The core modules of FedEnTrust include (1) heterogeneity-aware local model training, (2) knowledge distillation via soft label sharing, (3) blockchain-based secure coordination, and (4) adaptive ensemble aggregation.

These modules work together to realize 3 key objectives: maintaining patient privacy, enabling equitable participation across institutions with varying capacities, and ensuring secure collaboration in a decentralized system.

**Figure 1.**
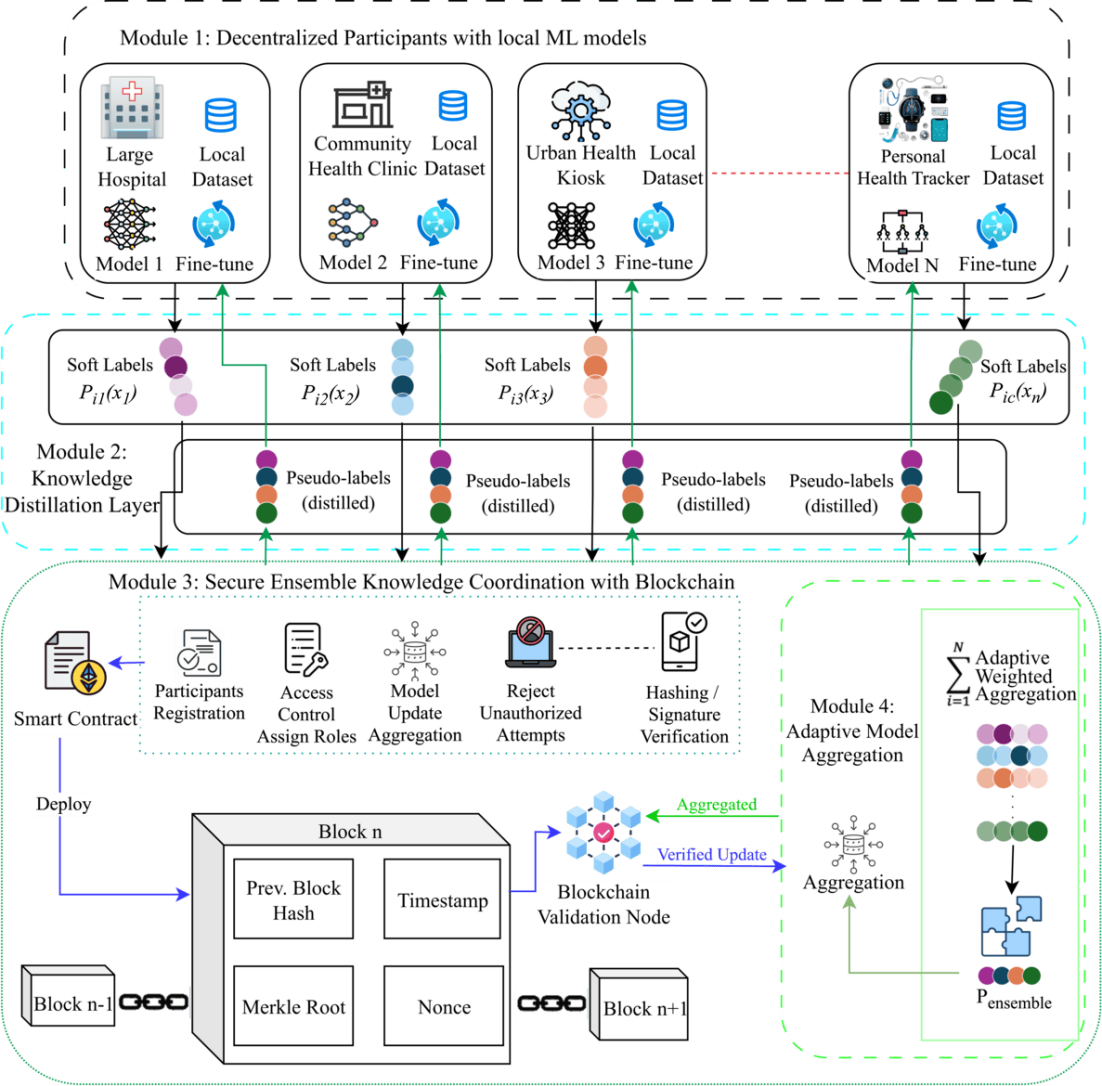
Overview of the FedEnTrust architecture. Soft labels generated by local models are authenticated through blockchain-based role-based access control and combined using adaptive weighted aggregation to produce pseudo labels for continued local training. ML: machine learning.

Figure 1 illustrates the end-to-end data flow across the 4 modules. Local raw data remain strictly on the device. Each participant trains a heterogeneous local model and generates soft-label probability vectors. These soft labels, along with accuracy metadata, are sent off-chain to the aggregator but must first pass through blockchain-based role-based access control (RBAC) validation, where the smart contract verifies participant identity, role permissions, and submission metadata. Validated soft labels are incorporated into an adaptive weighted aggregation mechanism, producing global pseudo-labels that are redistributed to all participants. The blockchain records transaction hashes and role enforcement events, ensuring traceability without revealing sensitive data.

### Architectural Novelty and Comparison With Existing FL Frameworks

Real-world health care environments exhibit substantial diversity in computational capacity, data distributions, trust requirements, and security risks. To contextualize the design of FedEnTrust within this landscape, we compare its architectural capabilities against representative FL and blockchain-enabled frameworks in Table 1.

**Table 1.** Architectural comparison of FedEnTrust with representative federated learning frameworks.

Challenge in real-world health care FL	FedEnTrust (Our work)	Hasan et al [15]	Biscotti [19]	Chang et al [20]	Microcontroller FL^a^ [17]
Heterogeneous compute environments (hospitals, clinics, kiosks, wearables)	Heterogeneity-aware model assignment; each node trains model matching its device capacity; ensemble aggregation aligns knowledge across disparate models	Supports ML^b^ models but generally assumes similar capacity clients	Assumes all clients run comparable gradient-sharing deep models	Single model structure required; difficult for low-resource clients	Designed for ultra-low-power devices; not suitable for multitier health care
Non-IID^c^ and imbalanced data across institutions	Soft-label knowledge distillation + weighted aggregation improve cross-site generalization	Local models trained independently; static averaging struggles with non-IID distributions	Gradient aggregation without distillation; non-IID data reduces convergence	DP^d^-sanitized gradients reduce signal strength on non-IID data	Very limited support for complex non-IID medical data
Cross-institution trust and secure participation	Smart contract–driven RBAC^e^; on-chain validation of model submissions; rejects malicious or unauthorized updates	Minimal security; no on-chain validation	Uses blockchain only as consensus layer, not for role-level access control	Smart contract manages DP gradients, not participation permissions	No trust or participation assurance mechanism
Protection against malicious updates (poisoning, fake uploads)	On-chain validator roles + metadata checks prevent poisoned soft labels before aggregation	No defense against malicious gradient or model uploads	Consensus prevents tampering but not model poisoning	DP reduces leakage but not poisoning	No adversarial defense features
Interoperability across model types	Soft labels unify outputs of RF^f^, XGB^g^, DT^h^, SVM^i^, KNN^j^ into comparable probability space	Homogeneous ML models; limited interoperability	Requires same model structure for gradient fusion	Single-model FL; weights must match	No model interoperability
Scalability across distributed health care networks	Lightweight soft-label sharing reduces communication overhead and suits mixed-resource environments	Local model averaging; moderate scalability	Heavy blockchain consensus overhead limits scalability	DP gradient exchange increases bandwidth needs	Limited to microcontroller networks
Auditability and traceability for compliance (HIPAA^k^ or GDPR^l^)	Full on-chain audit log of registrations, updates, and permissions	Centralized coordination; limited auditability	All gradient updates stored on-chain—high cost	Stores only gradient summaries; limited audit transparency	Not designed for regulated health care settings

a FL: federated learning.

b ML: machine learning.

c IID: independent and identically distributed.

d DP: differential privacy.

e RBAC: role-based access control.

f RF: random forest.

g XGB: extreme gradient boosting.

h DT: decision tree.

i SVM: support vector machine.

j KNN: k-nearest neighbors.

k HIPAA: Health Insurance Portability and Accountability Act.

l GDPR: General Data Protection Regulation.

Unlike approaches such as Hasan et al [15], Biscotti [19], and Chang et al [20], which rely on homogeneous model structures or gradient-based updates, FedEnTrust supports heterogeneity-aware model assignment. Each participant trains a locally suitable model (eg, random forest, extreme gradient boosting, decision tree, support vector machine [SVM], k-nearest neighbors [KNN]) based on its available resources, enabling participation from hospitals, clinics, kiosks, and wearable devices.

FedEnTrust also differs from blockchain-enabled systems such as Shalan et al [24] and TinyFL [25]. While these frameworks integrate blockchain for logging or access control, they do not incorporate soft-label knowledge distillation or adaptive ensemble aggregation to unify heterogeneous model outputs. FedEnTrust introduces a unique coupling of soft-label–based distillation with blockchain-enforced RBAC, enabling secure verification of participant identity and role prior to model update submission, on-chain logging of update hashes to ensure auditability, prevention of malicious or unauthorized contributions before they influence aggregation, and interoperability of predictions across diverse model architectures.

This integration ensures that only authenticated, validated soft labels contribute to the global model. This design is particularly effective for non-IID and imbalanced health care data settings, where traditional gradient-averaging approaches struggle.

### Module 1: Decentralized Local Training With Heterogeneous Models

FedEnTrust begins with a network of decentralized health care participants, including large hospitals, regional clinics, kiosks, and personal health trackers, each training its own machine learning model locally. These models are tailored to each participant’s computational capabilities and data volume. For example, high-resource hospitals may use deep neural networks, while low-resource settings use shallow learning such as KNN or support vector classifier (SVC) to support real-time inference with minimal memory demands.

This heterogeneity-aware model assignment ensures that all participants, regardless of scale or technical capacity, can contribute meaningfully. Local training is performed privately using internal datasets, aligning with privacy regulations such as HIPAA and GDPR.

### Module 2: Knowledge Distillation via Soft Labels

To facilitate collaborative learning without exposing raw data, participants generate soft labels, probability distributions over prediction classes (eg, diabetic, nondiabetic). These soft labels encode richer information than binary outputs and are shared with a central aggregator, enabling cross-site knowledge transfer.

#### Soft Label Generation

Each participant generates soft labels, probability distributions reflecting its model’s confidence across classes, and transmits these predictions to the aggregator. Unlike gradient-based approaches, soft labels create an interoperable representation across heterogeneous model types. Before being used for ensemble aggregation, every soft label submission is paired with metadata including local validation accuracy, model identifier, and round number. For an input instance *x*, the participant’s model outputs a probability vector:


(1) $$P_{i}(x)=[p_{1},p_{2},\ldots ,p_{c}]\in R^{c},\;\text{where }\sum_{c=1}^{C}p_{c}=1$$

These soft labels encapsulate the model’s confidence across the Cclasses and support knowledge transfer without sharing raw patient data or internal model parameters.

To address differences in how heterogeneous models calibrate probability outputs, FedEnTrust applies temperature scaling, which smooths the probability distribution by dividing logits $z_{i}(x)$by a temperature parameter T:


(2) $$\begin{array}{c} P_{i}^{\left(t\right)}\left(x\right)=\text{s}\text{o}\text{f}\text{t}\text{m}\text{a}\text{x}\;\left(\frac{z_{i}\left(x\right)}{T}\right),\;T=2 \end{array}$$

A temperature of T=2 was selected because values greater than 1 produce smoother, less overconfident probability distributions, which improves the stability of aggregation across models with different calibration characteristics. A small temperature (eg, T=1) can lead to overly sharp probabilities that amplify noise, while excessively large values dilute useful predictive signals. Empirical testing showed that T=2 offers an optimal balance.

#### Dynamic Weight Updates Across Federated Rounds

Once soft labels are generated by each participant model, the system proceeds to combine these distributed outputs into a unified global prediction. This ensemble consensus represents a key step in transferring collective intelligence across all nodes while respecting the constraints of data privacy and computational diversity.

 The ensemble aggregation process employs adaptive weighted soft voting, where more reliable and accurate models are given stronger influence. For example, a well-resourced clinic with consistently high validation performance will contribute more to the global prediction than a basic kiosk with limited data. However, no participant is excluded; each contributes according to its validated strength, ensuring fairness and inclusivity in the learning process. FedEnTrust adaptively updates the influence of each participant during communication round t. Each participant evaluates its model using a shared public validation subset to compute ${\text{Acc}}_{i}^{\left(t\right)}$, which is the validation accuracy of participant i at round t. The ensemble assigns each participant a normalized contribution weight:


(3) $$\begin{array}{c} W_{i}^{\left(t\right)}=\frac{{\text{Acc}}_{i}^{\left(t\right)}}{\sum_{j=1}^{N}{\text{Acc}}_{j}^{\left(t\right)}} \end{array}$$

To prevent dominant institutions (eg, large hospitals with more data) from exerting disproportionate influence, FedEnTrust applies weight clipping, capping $W_{i}^{\left(t\right)}$at an upper bound. This ensures contribution fairness and reduces the risk of bias toward specific demographic subpopulations.

#### Justification for Heterogeneous Model Assignment

The model architectures listed in Table 2 were selected to reflect realistic resource constraints and deployment contexts:

- Random forest (hospitals): Hospitals possess sufficient computational capacity and large datasets; random forest models capture nonlinear relationships and perform well on tabular clinical data.
- XGB (regional clinics): XGB provides strong performance under moderate computational resources, making it suitable for mid-sized clinics.
- Decision trees and KNN (community clinics or kiosks): These models require minimal training cost and support real-time inference in low-power environments.
- Linear SVM (wearables or personal trackers): Linear SVM has a lower memory footprint than logistic regression and offers more stable performance on small, noisy physiological samples typically produced by wearables.

**Table 2.** Simulated decentralized participants and their models.

ID	Participant	Model architecture	Key parameters	Resource level	Weight	Remarks
1	Large hospital	Random forest	n_estimators=130max_depth=15max_features=0.75 data_use=50%	Very high	0.50	Trains complex models on large datasets; serves as a high-capacity node
2	Urban health kiosk	K-nearest neighbors	n_neighbors=5 algorithm='auto'data_use=5%	Low	0.05	Designed for low-resource environments using simple, efficient models
3	Regional clinic	XGBoost	learning_rate=0.01max_depth=10n_estimators=180data_use=30%	High	0.30	Supports moderately complex modeling on medium-sized datasets
4	Community health clinic	Decision tree	max_depth=Nonecriterion='gini'data_use=10%	Medium	0.10	Runs interpretable tree-based models with moderate resource needs
5	Personal health tracker	Support vector machine	kernel='linear'C=1.0data_use=5%	Very low	0.05	Uses lightweight models suitable for wearables and embedded devices

This heterogeneity-aware mapping allows each participant to train a model aligned with its resource profile while still contributing to a unified ensemble.

#### Enhanced Knowledge Distillation and Pseudo-Label Generation

In each communication round t, participant models generate calibrated soft probability vectors $P_{i}^{t}(x)$, which are aggregated using dynamically updated participant weights to produce a global soft prediction.

Our proposed model aggregates the calibrated soft labels using the dynamic weights to produce a global soft prediction:


(4) $$\begin{array}{c} P^{t}\left(x\right)=\sum_{i=1}^{N}W_{i}^{t}*P_{i}^{t}\left(x\right) \end{array}$$

Because aggregation operates entirely on probability distributions rather than gradients or model parameters, FedEnTrust naturally supports heterogeneous machine learning architectures across hospitals, clinics, kiosks, and personal wearable devices while preserving data locality and privacy.

To improve the reliability of knowledge transfer, each participant’s soft predictions undergo normalization followed by temperature scaling (with T=2) to smooth overconfident outputs. The ensemble output is then evaluated using a confidence-based filtering mechanism, where pseudo-labels are generated only if the maximum ensemble probability satisfies:


(5) $$\begin{array}{c} \max\left(P^{t}\left(x\right)\right)\geq \tau \end{array}$$

With τ=0.7 Predictions failing this criterion are discarded to prevent the propagation of uncertainty or noise. Accepted pseudo-labels are normalized and redistributed to participants, where they are appended to local datasets and used for continued training in the subsequent round. This feedback loop enables low-resource participants to benefit from globally distilled knowledge while retaining local autonomy.

All soft-label submissions are validated through the blockchain-based RBAC mechanism described in Module 3. Only soft labels originating from authenticated and authorized roles (eg, model-provider) are accepted. Validated submissions are incorporated into an adaptive weighted soft-voting process, where participant weights are updated based on observed local performance across rounds. The resulting global outputs are then redistributed as pseudo-labels for the next training iteration, ensuring robustness against non-IID data distributions, preventing malicious or fabricated updates, and enhancing cross-site generalization across heterogeneous health care environments.

### Module 3: Blockchain-Based Secure Coordination

#### Overview

Module 3 employs an Ethereum-based smart contract to authenticate participants, enforce role permissions, and log immutable update metadata. When a node attempts to upload soft labels, the smart contract verifies the participant’s role, identity, timestamp, and declared accuracy. The contract then generates and stores a hashed representation of the update, which validator nodes review. Only soft labels that receive approval from multivalidators are admitted to the aggregation pool. This ensures tamper resistance, prevents poisoning attacks, and provides end-to-end traceability for health care compliance requirements. When a participant attempts to contribute soft labels, the smart contract performs the following checks:

1. Identity verification: Confirms that the contributor is a registered network participant.
2. Role validation: Ensures the contributor holds a permitted role to submit model outputs.
3. Metadata verification: Confirms the integrity of reported metrics (eg, accuracy, round number).
4. Hash logging: Stores a transaction hash to provide auditability without exposing any data.

Only after passing these checks is the soft label included in the aggregation pool. This design prevents poisoned or fabricated updates from influencing the global model and eliminates single points of failure in participation management. By integrating RBAC directly with knowledge distillation, FedEnTrust establishes a secure and transparent trust layer that coordinates collaborative learning across diverse health care nodes.

#### Blockchain Platform Selection and Justification

FedEnTrust is implemented on an Ethereum-compatible private blockchain network. Ethereum was selected due to its deterministic smart contract execution, robust security guarantees, and mature tooling ecosystem. The platform supports Solidity-based smart contracts, Remix IDE integration, and widely adopted standards for access control and event logging. These characteristics make Ethereum well suited for privacy-preserving health care collaboration, where verifiable execution and auditability are required.

To justify this choice, we compared Ethereum with 2 commonly used permissioned blockchain platforms: Hyperledger Fabric and Corda. Table 3 presents a feature-level comparison of Ethereum, Hyperledger Fabric, and Corda across network type, decentralization, smart contract support, privacy mechanisms, ecosystem maturity, and application alignment. Given the need for flexible smart contract logic, verifiable coordination, and broad compatibility with Internet of Things (IoT) and health care prototypes, Ethereum provides the most practical platform for FedEnTrust.

**Table 3.** Comparison of blockchain platforms.

Feature	Ethereum	Hyperledger fabric	Corda
Network type	Public or private	Permissioned	Permissioned
Decentralization	Highly decentralized	Semi-decentralized	Semi-decentralized
Smart contracts	Solidity, robust tooling	Chaincode (Go/Java/Node.js)	Contract flows for financial logic
Privacy	Extensible via Layer-2/private networks	Strong privacy (channels, private collections)	Strong bilateral privacy
Ecosystem	Very large developer ecosystem	Enterprise-focused	Financial institutions
Use alignment	Decentralized coordination across heterogeneous nodes	Consortium-style enterprise networks	Regulated financial workflows

#### Adversarial Threat Model and Security Resilience

FL deployments in real-world health care environments may be exposed to adversarial participants attempting to manipulate the global model, disrupt training, or infer sensitive information. To address these risks, we construct a structured threat model covering three primary attack categories: (1) model poisoning; (2) collusion among compromised participants; and (3) malicious soft-label injection, where adversaries submit manipulated pseudo-probabilities to bias the aggregation process.

FedEnTrust incorporates multiple, tightly coupled defense mechanisms across its blockchain coordination and ensemble aggregation layers to provide resilience against these threats.

1. Model poisoning and malicious soft-label injection: A compromised participant may attempt to submit adversarial or fabricated soft labels to influence global predictions. FedEnTrust mitigates this risk through smart contract–enforced RBAC, which restricts update submission exclusively to authenticated participants holding an authorized model-provider role. Each submission is accompanied by metadata including round number, reported validation accuracy, and timestamp, which are verified for internal consistency before acceptance. To ensure integrity and prevent replay or tampering, all submissions are cryptographically hashed and logged on-chain. Furthermore, FedEnTrust employs validator redundancy, requiring approval from multiple trusted validator nodes (eg, lead hospitals within the consortium) before a submission is incorporated into aggregation, preventing single-node compromise.
2. Collusion and validator compromise: To reduce the impact of colluding or compromised participants, FedEnTrust adopts a consortium-style multivalidator approval mechanism. No single validator can independently approve a model update; instead, a quorum of validators must jointly authorize submissions. The validator set itself is managed through governed smart contract functions, allowing secure updates to validator membership over time and eliminating static trust assumptions.
3. Blockchain-specific threats: Public blockchain deployments may be vulnerable to front-running, transaction reordering, or gas manipulation attacks. FedEnTrust avoids these risks by operating on a private Ethereum-compatible consortium network without a public mempool, eliminating front-running opportunities. Smart contracts use fixed gas budgets and sequential transaction counters to ensure deterministic execution and prevent reordering attacks.
4. Privacy leakage through on-chain metadata: Although raw data and model parameters are never shared, metadata leakage can still pose privacy risks. FedEnTrust minimizes exposure by storing only hashed identifiers and role-verification logs on-chain. No patient-level attributes, raw predictions, or model parameters are recorded. All soft labels remain strictly off-chain and are exchanged only between authorized participants and the aggregator over secure channels.
5. Aggregation-level safeguards: Beyond blockchain enforcement, the adaptive ensemble layer further mitigates adversarial influence by applying temperature scaling, confidence thresholds, and weight clipping. These mechanisms limit the amplification of extreme or adversarial soft-label probabilities and restrict the maximum influence any single participant can exert, even if it reports high accuracy.

Collectively, these mechanisms establish a multilayered security architecture that protects FedEnTrust against common poisoning, collusion, and manipulation attempts at the coordination and authorization layers while preserving decentralized operation and data privacy. The empirical results demonstrate that unauthorized and malicious submissions are consistently detected and rejected through blockchain-enforced RBAC and validator checks. While this study focuses on secure enforcement and system robustness rather than controlled adversarial learning simulations, the framework is explicitly designed to support future evaluation against targeted and untargeted attacks, including label-flipping, probability-shifting, and adaptive adversarial strategies.

### Module 4: Adaptive Model Aggregation and Feedback Loop

After soft labels are aggregated into a global ensemble prediction, FedEnTrust redistributes this consensus to participants as pseudo-labels for retraining. This adaptive aggregation ensures that high-performing models contribute more to the global prediction, while low-resource nodes still benefit from the collective knowledge.

This module enables faster convergence across non-IID data, fair and inclusive participation, and improved generalization without data sharing.

The result is a balanced feedback loop: local models become more aligned with the ensemble, improving personalization and global performance over time.

### System Implementation and Evaluation Setup

We evaluated FedEnTrust using the publicly available PIMA Indians Diabetes Dataset [26], which includes 768 records of female patients with 8 clinical attributes and a binary diabetes outcome. Data were preprocessed using the following steps:

1. Outlier detection with IQR and local outlier factor
2. Feature engineering (eg, binning glucose, insulin levels)
3. Normalization using *z* scores
4. Class balancing using the synthetic minority oversampling technique [27]

As shown in Table 1, to simulate a real-world heterogeneous environment, the dataset was split across 5 simulated participants with varying data volumes and models. Each participant’s computational weight was reflected in the aggregation process, mimicking operational conditions ranging from large hospitals to low-power personal devices.

### Ethical Considerations

This study exclusively used publicly available, deidentified secondary datasets. No new data were collected, and no interaction with human participants occurred. According to institutional policy and US federal regulations (45 CFR 46), research involving publicly available, deidentified data does not constitute human participant research and is therefore exempt from institutional review board review. As a result, institutional review board approval was not sought, and informed consent was not required. All datasets used in this study were fully deidentified prior to public release. The data contained no direct or indirect identifiers, and no attempt was made to reidentify individuals. Data were accessed and analyzed in accordance with the terms and conditions specified by the data providers. No participants were recruited for this study, and no compensation was provided.

## Results

### Model Performance

We evaluated the FedEnTrust framework across 5 heterogeneous participants over 15 communication rounds, focusing on prediction accuracy, precision, recall, and *F*_1_-score. The results highlight how collaborative learning and adaptive aggregation significantly enhance performance, especially for participants with limited data and computational resources.

**Figure 2.**
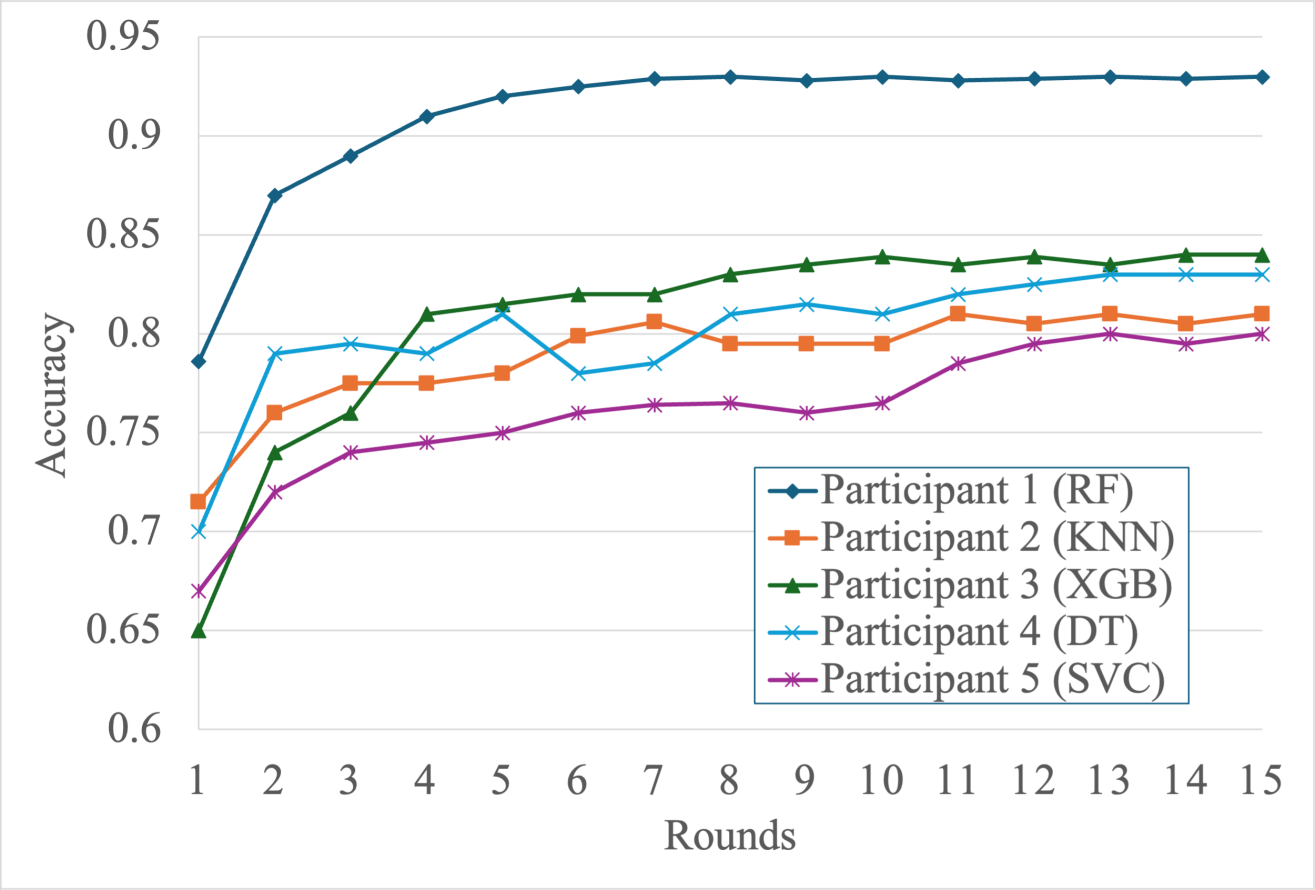
Global model accuracy improves over ensemble federated round. DT: decision tree; KNN: k-nearest neighbors; RF: random forest; SVC: support vector classifier; XGB: extreme gradient boosting.

Figure 2 shows the accuracy trajectories of each participant over the FL rounds. Participant 1 (random forest), equipped with the largest dataset and the highest computational power, consistently achieved the highest accuracy, acting as a de facto “teacher” during knowledge distillation. Its influence helped guide improvements in lower-resource nodes, such as participant 5 (SVC) and participant 2 (KNN), which showed steady gains over time.

**Figure 3.**
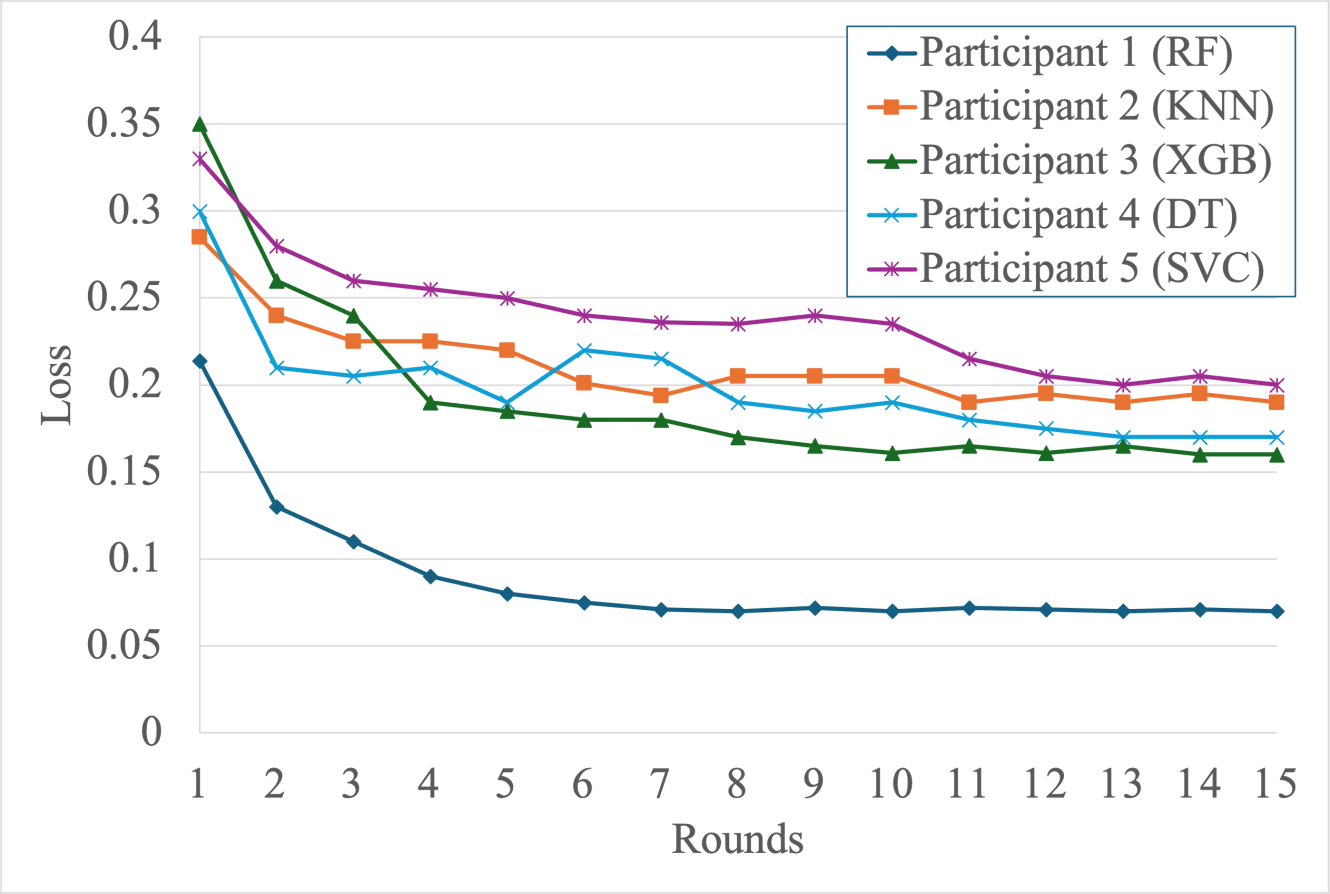
Federated model losses over rounds. DT: decision tree; KNN: k-nearest neighbors; RF: random forest; SVC: support vector classifier; XGB: extreme gradient boosting.

Figure 3 presents the corresponding model loss curves. All participants experienced substantial loss reduction early on, with convergence observed by round 15. Participant 1 maintained the lowest loss throughout, while participants 4 and 5 showed marked improvement from higher initial losses, demonstrating the benefit of federated collaboration.

Table 5. Federated Models Performance after 15 rounds

Comparing the initial and federated performance results (Tables4  5) reveals substantial gains for all participants after collaborative training. Accuracy improvements of up to 28% are observed in lower-resource participants, and *F*_1_-scores increase consistently across all models, demonstrating the effectiveness of knowledge distillation and adaptive aggregation in heterogeneous environments. For example, participant 4 (decision tree) improves its *F*_1_-score from 0.71 to 0.88, while participant 3 (XGBoost) improves from 0.64 to 0.85, highlighting the benefits of ensemble-driven knowledge transfer.

**Table 4.** Initial models’ performance.

Participant	Accuracy	Precision	Recall	*F*_1_-score
1	0.78	0.85	0.84	0.83
2	0.71	0.73	0.71	0.72
3	0.65	0.63	0.65	0.64
4	0.70	0.73	0.71	0.71
5	0.67	0.67	0.68	0.67

**Table 5.** Federated models’ performance after 15 rounds.

Participant	Accuracy	Precision	Recall	*F*_1_-score
1	0.93	0.92	0.94	0.93
2	0.81	0.80	0.86	0.83
3	0.84	0.85	0.86	0.85
4	0.83	0.87	0.90	0.88
5	0.80	0.79	0.87	0.83

To further characterize performance stability across communication rounds, Table 6 reports both the final accuracy at round 15 and the mean (SD) of accuracy over all 15 federated rounds. The relatively low SDs indicate stable convergence behavior for all participants, even for lightweight models such as KNN and SVC. These results confirm that FedEnTrust effectively accommodates device and data heterogeneity while maintaining strong predictive performance, privacy preservation, and decentralized operation. Tailored model architectures, aligned with participant resource constraints, ensure balanced contribution and efficient deployment across the collaborative learning process.

**Table 6.** Federated model accuracy and variability across 15 rounds.

Participant	Model	Final accuracy	Accuracy, mean (SD)
1	RF^a^	0.93	0.91 (0.04)
2	KNN^b^	0.81	0.79 (0.03)
3	XGB^c^	0.84	0.81 (0.05)
4	DT^d^	0.83	0.80 (0.03)
5	SVC^e^	0.80	0.76 (0.03)

a RF: random forest.

b KNN: k-nearest neighbors.

c XGB: extreme gradient boosting.

d DT: decision tree.

e SVC: support vector classifier.

To assess whether the performance differences between FedEnTrust and baseline models were statistically meaningful on the PIMA Indians Diabetes Dataset, we conducted a nonparametric bootstrap significance analysis using the same held-out test set as the main evaluation. Because accuracy, precision, recall, and *F*_1_-score are bounded metrics that may deviate from normality, bootstrap resampling provides a distribution-free and robust alternative to parametric methods such as the *t* test. We used a 2-tailed *t* test, as no directional assumption was imposed a priori and the objective was to assess whether there was any statistically significant difference between the compared methods.

We generated B=1000 bootstrap resamples by sampling test instances with replacement from the held-out evaluation set. For each bootstrap resample, we evaluated FedEnTrust and the decentralized baseline from Blockchain-FL with Differential Privacy [20], which represents the closest methodologically comparable prior work under similar privacy and decentralization constraints. This procedure produced 1000-sample empirical distributions for both models’ accuracy. To quantify comparative performance, we computed the bootstrap metric difference for each resample:


(6) $$\begin{array}{c} \Delta^{\left(b\right)}=M_{\text{FedEnTrust}}^{\left(b\right)}-M_{\text{Baseline}}^{\left(b\right)} \end{array}$$

where $M^{b}$ represents the accuracy, precision, recall, or *F*_1_-score on bootstrap resample b. We then constructed 95% CIs for each metric difference using the percentile method.

The bootstrap CI analysis indicates that FedEnTrust achieves statistically significant performance improvements over the decentralized blockchain-based FL baseline [20]. Specifically, FedEnTrust attains a mean accuracy of 0.842 with a 95% bootstrap CI of 0.831-0.853, compared to 0.827 (0.814-0.839) for the decentralized baseline. The resulting accuracy difference of +0.015 yields a 95% CI of 0.004-0.027, which excludes zero, indicating statistical significance at *α*=.05. These results confirm that the performance gains observed for FedEnTrust are not due to random variation but rather stem from its integration of heterogeneous ensemble learning with blockchain-backed coordination under privacy constraints.

These findings validate that FedEnTrust’s performance gains are not only empirical but statistically robust, reinforcing the effectiveness of combining heterogeneous ensemble learning with blockchain-backed coordination in constrained health care environments.

### Blockchain Performance

We deployed the smart contract with 6 key functions and evaluated it under a realistic configuration consisting of 5 decentralized health care participants and 1 global aggregator. These components facilitated secure collaboration, access management, and federated training. The details are shown in Table 7.

**Table 7.** Blockchain system configuration.

Operation	Count	Description
Total registered participants	5	Registered using registerClient()
Federated coordination nodes	1	Global aggregator for accuracy aggregation and model ensemble
Smart contract functions deployed	6	Includes registration, role assignment, update logging, and access checks

To assess computational efficiency, we monitored key metrics such as gas consumption, data size, and latency for major smart contract operations. These measurements reflect the cost-effectiveness and responsiveness of blockchain-mediated tasks.

These operations incur gas overhead beyond Ethereum’s 21,000 base gas due to additional computation, state updates, and event emissions. The *modelUpdate*() function, for example, consumes about 98,560 gas (~295 bytes of encoded parameters), balancing cost with functional depth and traceability (Table 8).

**Table 8.** Smart contract performance metrics.

Operation	Average gas cost	Data size (bytes)	Average latency (ms)
Client registration	118,073	370	220
Role assignment	109,820	345	210
Model update	98,560	295	195
Model aggregation	105,310	315	215

Despite slight delays compared to traditional systems, the observed latency (195‐220 ms) remains acceptable for health care applications, considering the gains in trust, verifiability, and tamper resistance. To assess longer-term stability, we analyzed all 212 smart contract operations recorded during the training. All valid transactions executed successfully without anomalies, indicating stable performance across repeated interactions. The expanded evaluation in Table 9 includes average latency, latency range, and variability across extended cycles. These findings support the suitability of the blockchain layer for multiround federated training.

**Table 9.** Transaction integrity and enforcement metrics.

Category	Values	Description
Total transactions	212	All smart contract operations
Valid transactions	201	Successfully executed by authorized participants
Rejected transactions	11 (5.19%)	Unauthorized queries (6), malicious submissions (3), invalid role updates (2)
Success rate	100%	All valid transactions completed without error
Average latency	21.4 ms	Mean execution time for valid transaction
Latency range	14.8‐36.2 ms	Minimum and maximum observed latency
SD	±4.7 ms	Variability in execution time
Latency over extended cycles (100 iterations)	Mean: 22.1 ms; variation:±5.3 ms	Long-term stability testing simulating multiround FL^a^
Finality time	~1 block(~1 s)	Deterministic finality in private PoA^b^ Ethereum network
Estimated throughput	~47 tx/s	Consistent with private Ethereum networks

a FL: federated learning.

b PoA: proof-of-authority.

**Figure 4.**
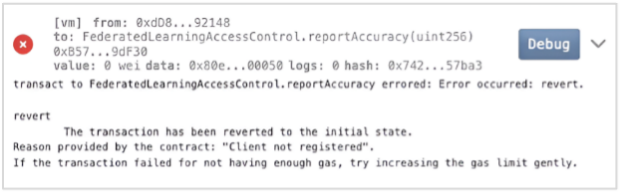
Access rejection for unauthorized participant.

As illustrated in Figure 4, unauthorized model submissions are automatically rejected, triggering an on-chain error: “Client not registered.” This ensures that only authenticated nodes contribute to the learning process, strengthening data integrity.

Throughout 15 communication rounds, the smart contract reliably supported secure, real-time exchange of soft label predictions and model aggregation updates. For instance, participant 1 improved from 78% to 93% accuracy, while participant 4 rose from 70% to 83%, all while maintaining privacy and resisting tampering.

These results underscore the effectiveness of combining blockchain with federated ensemble learning to achieve scalable, secure, and privacy-preserving AI in health care environments.

## Discussion

### Principal Findings

This study presents FedEnTrust, a blockchain-enabled federated ensemble learning framework that offers a privacy-preserving and scalable solution for decentralized diabetes prediction. Our system effectively balances accuracy, privacy, and adaptability by integrating diverse machine learning models with knowledge distillation and adaptive weighted aggregation. With a predictive accuracy of 84.2%, FedEnTrust demonstrates competitive performance while maintaining strict privacy guarantees and supporting heterogeneous health care participants ranging from hospitals to wearable devices.

The framework’s integration with blockchain smart contracts provides secure participant coordination, role-based access control, and transparent model validation without incurring substantial latency or resource overhead. Importantly, our results show that even low-resource participants benefit from collaboration through soft label exchange, enabling equitable participation in the learning process.

### Comparison With Prior Work

Table 10 summarizes the performance of FedEnTrust against the existing centralized and decentralized methods applied to the PIMA Indians Diabetes Dataset. While centralized deep learning approaches achieve slightly higher accuracy (eg, 95.2% with light gradient boosting machine, 96.1% with convolutional neural networks), these models require full data centralization, sacrificing privacy and increasing system vulnerability.

**Table 10.** Comparative performance on the PIMA Indians Diabetes Dataset.

Model or study	Accuracy (%)	Precision (%)	Recall (%)	*F*_1_-score (%)	Notes
FedEnTrust	84.2	84.6	88.6	86.4	Federated ensemble with adaptive weighted voting and blockchain smart contract integration
ML^a^ classifiers approach [28]	95.2	N/A^b^	N/A	N/A	Centralized; evaluated multiple classifiers (LR^c^, XGB^d^, GB^e^, DT^f^, ET^g^, RF^h^, and LGBM^i^) on PIMA Indians dataset; best accuracy achieved by LGBM
Recursive feature elimination with a gated recurrent unit RFE-GRU^j^ [29]	90.7	90.5	90.7	90.5	Centralized; utilized RFE-GRU on PIMA Dataset
Hybrid classification approach [30]	83.1	N/A	64.8	N/A	Centralized; applied SVM^k^, RF, DT, naive Bayes with K-means preprocessing; best accuracy achieved by SVM
Three predictive algorithms [31]	77.1	N/A	N/A	N/A	Centralized; applied LR, RF, and ANN^l^; LR achieved the best accuracy (77.10%) with AUC^m^ 0.83 over RF and ANN
Soft voting ensemble [32]	79.1	73.1	71.6	80.9	Centralized; combined RF, LR, and naive Bayes classifiers
Ensemble hierarchical model [33]	83.1	25.0 (positive)/98.6 (negative)	38.4 (positive)/90.2 (negative)	82.8	Centralized; applied DT and LR, fused by neural network
Stacking ensemble [25]	77.1	N/A	N/A	N/A	Centralized; stacking ensemble of ML models; accuracy achieved using cross-validation protocol
Deep learning pipeline [34]	92.3	N/A	N/A	N/A	Centralized; deep learning pipeline using VAE^n^ for data augmentation, SAE^o^ for feature augmentation, and CNN^p^ for classification
Deep CNN with correlation-based features [35]	96.1	94.4	94.4	94.5	Centralized; applied deep CNN with feature selection based on correlation
Blockchain-FL with adaptive DP [20]	82.7	N/A	N/A	N/A	Decentralized; implemented federated learning with differential privacy using blockchain technology

a ML: machine learning.

b N/A: not applicable.

c LR: logistic regression.

d XGB: extreme gradient boosting.

e GB: gradient boosting.

f DT: decision tree.

g ET: extra tree.

h RF: random forest.

i LGBM: light gradient boosting machine.

j RFE-GRU: Recursive Feature Elimination with Gated Recurrent Unit.

k SVM: support vector machine.

l ANN: artificial neural network.

m AUC: area under the curve.

n VAE: variational autoencoder.

o SAE: stacked autoencoder.

p CNN: convolutional neural network.

In contrast, FedEnTrust improves over recent decentralized models, such as blockchain-integrated FL with differential privacy (accuracy≈82.7%), by incorporating ensemble learning and adaptive aggregation. Despite the constraints of data fragmentation and heterogeneity, our framework maintains robust performance across all key metrics, including precision (84.6%), recall (88.6%), and *F*_1_-score (86.4%).

FedEnTrust achieves a favorable trade-off between privacy, generalizability, and computational practicality, making it well suited for real-world deployment in regulated health care environments.

### Ethical AI Considerations: Fairness, Transparency, and Accountability

#### Ethical Framework

Ethical concerns are central to the deployment of AI systems in health care, where unequal access to computational resources and imbalanced data distributions may inadvertently create or reinforce model biases. FedEnTrust incorporates several design principles aligned with emerging ethical AI guidelines, including those recommended by the World Health Organization and major AI governance frameworks.

#### Fairness Across Heterogeneous Participants

Health care institutions vary substantially in data volume, demographic composition, and computational capacity, which can introduce systematic bias in collaborative learning systems. FedEnTrust is designed to mitigate such bias by supporting heterogeneity-aware participation, allowing low-resource nodes to contribute using models aligned with their capabilities without sacrificing predictive performance. Adaptive weight clipping is applied during aggregation to prevent high-resource institutions from disproportionately dominating the global ensemble. In addition, temperature-calibrated soft labels are used to reduce overconfidence from models trained on larger or more homogeneous datasets, while confidence thresholding ensures that noisy or low-confidence predictions are not propagated across participants. Together, these mechanisms promote more balanced influence across diverse health care contributors and support fairer model outcomes in heterogeneous federated environments.

#### Transparency and Auditability

Transparency in FedEnTrust is enabled through the blockchain-based coordination layer, which provides immutable audit trails for all update submissions and verifiable records of role validation events. Each model contribution is traceably logged, allowing the system to record which institutions participated in and influenced each training round. This tamper-resistant logging mechanism enhances accountability, supports post hoc auditing, and increases trust among participating health care entities without exposing sensitive data or model parameters.

#### Privacy and Data Minimization

FedEnTrust adheres to privacy-by-design principles:

- Raw patient data remain strictly on the device
- Only soft-label vectors and hashed metadata are transmitted
- No identifiable information is stored on-chain, supporting HIPAA, GDPR, and similar regulatory frameworks

Role-based access ensures that only authorized clinical entities may participate.

#### Accountability and Governance

The multivalidator consensus layer enables shared governance rather than reliance on a single coordinating institution. This creates a more accountable decision-making process and aligns with ethical expectations for distributed medical AI systems.

### Blockchain Performance and Practical Considerations

#### Implementation Considerations

Beyond empirical accuracy and security validation, the practical deployment of blockchain-enabled FL systems in health care requires careful consideration of scalability, cost, and regulatory compliance. While the blockchain layer in FedEnTrust demonstrated stable and reliable performance under controlled experimental conditions, real-world health care environments introduce additional operational and governance challenges. This section discusses key practical considerations and outlines how FedEnTrust is designed to address them.

#### Scalability and Throughput

Public blockchain platforms, such as the Ethereum main net, face inherent constraints related to transaction throughput, block confirmation latency, and network congestion. These limitations can lead to unpredictable delays and may not support the repeated coordination required across multiple FL rounds. To address this, FedEnTrust is designed for deployment on private or consortium-based Ethereum networks, where consensus parameters, block times, and validator participation can be tailored to health care workflows. Such configurations enable deterministic execution and consistent performance, as observed in our evaluation. Nevertheless, large-scale deployments involving many institutions may require additional enhancements, including optimized validator load balancing, hierarchical or sharded blockchain structures, and integration with layer-2 scaling mechanisms to further increase throughput.

#### Cost Variability and Resource Requirements

In public blockchain environments, gas fees fluctuate dramatically based on network conditions, resulting in variable operational costs for smart contract execution. This variability is incompatible with cost-sensitive health care environments. Deploying FedEnTrust on a private Ethereum network eliminates transaction fees and allows institutions to control computational and storage overhead. However, operating such networks requires institutional commitment to maintain validator nodes, ensure uptime, and manage governance policies. Future work will investigate cost-benefit trade-offs between private, hybrid, and layer-2 blockchain configurations for FL.

#### Regulatory and Compliance Constraints

Health care systems must comply with strict privacy regulations such as HIPAA, GDPR, and provincial or national data-protection laws. These frameworks introduce challenges, such as prohibiting the storage of patient data or identifiers on-chain, requiring transparent audit trails for collaborative analytics, and ensuring that cross-institution coordination adheres to data-sharing agreements.

FedEnTrust addresses these concerns by storing only hashed metadata and role-verification entries on-chain, keeping soft labels and model outputs entirely off-chain. However, real-world deployment requires integration with institutional governance mechanisms to ensure compliance documentation, legal interoperability among institutions, and formal auditing procedures.

### Generalizability to Multimodal and Longitudinal Health Care Data

Although the PIMA dataset provides a controlled benchmark for evaluating prediction accuracy, it does not reflect the complexity of real-world clinical environments. Modern health care systems generate multimodal data that may include structured electronic health record fields, laboratory values, medical imaging, clinician notes, and continuous wearable sensor streams. Additionally, many health conditions, including diabetes, require longitudinal modeling to capture evolving physiological states over time.

FedEnTrust is designed to naturally extend to these scenarios. The framework’s heterogeneity-aware model assignment allows each participant to select model architectures aligned with its data modality and computational resources. For example, hospitals could train sequence models (eg, long short-term memories or transformers) on longitudinal EHR data, while wearable devices may contribute short-term physiological features via lightweight SVM or tree-based models. The knowledge-distillation component operates on probability distributions and is therefore agnostic to model type, enabling soft-label fusion across diverse modalities and temporal structures. This capability is particularly suitable for integrating outputs from time-series models, tabular models, and sensor analytics.

The blockchain-based coordination layer also supports generalization, as its role-based validation and update logging apply to any model output regardless of modality. Future work will apply FedEnTrust to multicenter datasets such as MIMIC-IV, NHANES, and integrated wearable–EHR cohorts to evaluate its performance under more heterogeneous and clinically realistic conditions.

### Limitations

Despite promising results, several limitations remain:

- Dataset representativeness: The PIMA dataset is limited in scope and population diversity. Future work should evaluate FedEnTrust on broader, real-world datasets from varied demographics and geographies.
- Extreme client heterogeneity: Devices with ultra-low resources may still face difficulties in real-time model adaptation. Exploring ultra-lightweight architectures and communication compression techniques is a key next step.
- Controlled blockchain simulation: Our blockchain operations were simulated under stable conditions. Future deployment on public testnets or mainnets is necessary to assess real-world transaction delays, scalability, and cost variability.
- Advanced threat modeling: While the smart contract blocks unauthorized actions, adversarial behaviors such as collusion or model poisoning were not addressed. Future extensions may integrate anomaly detection and audit trails to enhance system resilience.

Although the PIMA Indians Diabetes Dataset is a well-established benchmark for evaluating diabetes prediction models, its limited demographic diversity and relatively small size restrict the generalizability of the findings. The simulated heterogeneous environment in Table 2, while constructed to reflect realistic participant variability, does not fully replicate the complexity of multi-institution health care settings, where differences in clinical practice, sensor characteristics, and patient demographics lead to substantially wider non-IID distributions. Accordingly, the results presented here should be viewed as a controlled feasibility demonstration rather than a comprehensive real-world validation.

### Conclusions

This study presents FedEnTrust, a secure and intelligent federated ensemble learning framework for privacy-preserving diabetes prediction. Our approach addresses key challenges in decentralized health care AI, including data privacy, system trust, and participant heterogeneity, without requiring access to raw patient data.

By integrating knowledge distillation and adaptive ensemble aggregation, the framework enables resource-aware contributions from a diverse range of participants, from high-performance hospital systems to low-power personal devices. The experimental results demonstrate consistent improvements in predictive performance across all participants, validating both the effectiveness and inclusiveness of the design.

A central innovation is the blockchain-enabled coordination layer, which ensures secure registration, role-based access control, and verifiable model updates. Smart contract simulations confirm the system’s efficiency, low latency, and robustness against unauthorized actions, supporting scalable and tamper-resistant deployment in health care environments.

In sum, FedEnTrust offers a practical, scalable solution for secure, decentralized medical AI, balancing privacy, performance, and trust. Future work will extend this framework to additional clinical domains, multisite studies, and dynamic personalization for broader impact in real-world health care.

## Abbreviations

AI: artificial intelligence
FL: federated learning
GDPR: General Data Protection Regulation
HIPPA: Health Insurance Portability and Accountability Act
IID: independent and identically distributed
KNN: k-nearest neighbors
RBAC: role-based access control
SVC: support vector classifier
SVM: support vector machine
